# Assessing Progress, Impact, and Next Steps in Rolling Out Voluntary Medical Male Circumcision for HIV Prevention in 14 Priority Countries in Eastern and Southern Africa through 2014

**DOI:** 10.1371/journal.pone.0158767

**Published:** 2016-07-21

**Authors:** Katharine Kripke, Emmanuel Njeuhmeli, Julia Samuelson, Melissa Schnure, Shona Dalal, Timothy Farley, Catherine Hankins, Anne G. Thomas, Jason Reed, Peter Stegman, Naomi Bock

**Affiliations:** 1 Project SOAR (Supporting Operational AIDS Research), Avenir Health, Washington, District of Columbia, United States of America; 2 U.S. Agency for International Development, Washington, District of Columbia, United States of America; 3 World Health Organization, Geneva, Switzerland; 4 Project SOAR, Palladium Group, Washington, District of Columbia, United States of America; 5 Sigma3 Services, Nyon, Switzerland; 6 Amsterdam Institute for Global Health and Development, Amsterdam, the Netherlands; 7 Naval Health Research Center, US Department of Defense, San Diego, California, United States of America; 8 Jhpiego, Washington, District of Columbia, United States of America; 9 U.S. Centers for Disease Control and Prevention, Atlanta, Georgia, United States of America; Cardiff University, UNITED KINGDOM

## Abstract

**Background:**

In 2007, the World Health Organization and the Joint United Nations Programme on HIV/AIDS (UNAIDS) identified 14 priority countries across eastern and southern Africa for scaling up voluntary medical male circumcision (VMMC) services. Several years into this effort, we reflect on progress.

**Methods:**

Using the Decision Makers’ Program Planning Tool (DMPPT) 2.1, we assessed age-specific impact, cost-effectiveness, and coverage attributable to circumcisions performed through 2014. We also compared impact of actual progress to that of achieving 80% coverage among men ages 15–49 in 12 VMMC priority countries and Nyanza Province, Kenya. We populated the models with age-disaggregated VMMC service statistics and with population, mortality, and HIV incidence and prevalence projections exported from country-specific Spectrum/Goals files. We assumed each country achieved UNAIDS’ 90-90-90 treatment targets.

**Results:**

More than 9 million VMMCs were conducted through 2014: 43% of the estimated 20.9 million VMMCs required to reach 80% coverage by the end of 2015. The model assumed each country reaches the UNAIDS targets, and projected that VMMCs conducted through 2014 will avert 240,000 infections by the end of 2025, compared to 1.1 million if each country had reached 80% coverage by the end of 2015. The median estimated cost per HIV infection averted was $4,400. Nyanza Province in Kenya, the 11 priority regions in Tanzania, and Uganda have reached or are approaching MC coverage targets among males ages 15–24, while coverage in other age groups is lower. Across all countries modeled, more than half of the projected HIV infections averted were attributable to circumcising 10- to 19-year-olds.

**Conclusions:**

The priority countries have made considerable progress in VMMC scale-up, and VMMC remains a cost-effective strategy for epidemic impact, even assuming near-universal HIV diagnosis, treatment coverage, and viral suppression. Examining circumcision coverage by five-year age groups will inform countries’ decisions about next steps.

## Introduction

In March 2007, the World Health Organization (WHO) and the Joint United Nations Programme on HIV/AIDS (UNAIDS) recommended male circumcision (MC) as an additional method of HIV prevention, and urged countries with low MC prevalence and generalized HIV epidemics to rapidly scale up voluntary medical male circumcision (VMMC) programs in the context of combination prevention. Acting on this recommendation, several countries set up programs that offered VMMC to males requesting the procedure. These programs included promotion of condoms and safer sexual practices, treatment for sexually transmitted infections, and HIV testing and counseling with linkage to HIV care and treatment for those diagnosed with HIV.

Several mathematical models estimated the impact on the HIV epidemic of scaling up VMMC programs in diverse African regional settings (sub-Saharan Africa, southern Africa, and eastern Africa) and specific countries (Botswana, Kenya, South Africa, and rural Uganda). A consensus meeting in 2008 examined the models and generated answers to key policy questions on VMMC scale-up [1]. This led to the development of a detailed impact and costing model called the Decision-Makers’ Program Planning Tool (DMPPT), designed to help national policy makers decide the scope and pace of their country’s VMMC scale-up.

In 2011, the DMPPT was used to model the impact and cost of VMMC scale-up in 13 high-priority countries in eastern and southern Africa with high HIV incidence and low MC coverage (Botswana, Lesotho, Malawi, Mozambique, Namibia, Rwanda, South Africa, Swaziland, Tanzania, Uganda, Zambia, Zimbabwe, and Nyanza Province, in Kenya) [2]. The model estimated that 20.3 million circumcisions would be required to increase circumcision prevalence from 2011 baseline levels to 80% by the end of 2015 in men ages 15–49 years. It predicted that if 80% male circumcision prevalence was then maintained through 2025 (requiring an additional 8.4 million circumcisions over 10 years), a total of 3.36 million HIV infections would be averted over the period 2011–2025, representing 22% of expected new HIV infections. Based on limited pilot program data, the estimated median cost per circumcision was $83 USD (range $66–$95 USD), leading to an estimated median cost of $700 USD (range $370–$4,100 USD) per infection averted. (All subsequent references to currency are in U.S. dollars.) Compared with an estimated lifetime HIV treatment cost of $7,400, the model predicted excellent value for money when averted treatment costs were considered.

VMMC programs have now been implemented in the original 13 high-priority countries plus Ethiopia. In light of five years of accumulated implementation experience, a new mathematical model—the Decision Makers’ Program Planning Tool, Version 2 (DMPPT 2)—was created to address questions having to do with age and geographic prioritization. DMPPT 2 projects the impact, cost, and cost-effectiveness of VMMC scale-up disaggregated by five-year client age group and subnational region. It provides scenarios for continued expansion of VMMC programs to reach and maintain specified coverage targets by age group, and incorporates new HIV incidence estimates from country surveillance data collected since 2010. Elsewhere in this collection, we describe this model [3], along with five country model applications [4–8].

By the end of 2014, the 14 countries had provided VMMC to more than 9 million men, nearly half way to the original target of 20 million [9]. Given lessons learned from implementation, new modeling studies, and changes in the HIV field, we thought it timely to reassess progress toward the targets and project the impact of the VMMCs conducted already through 2014. The future HIV incidence projections used in the model in this paper are informed by new surveillance data and assume scaling up antiretroviral therapy (ART) to reach the 90-90-90 treatment goals by 2020 proposed by UNAIDS [10]. (These goals stipulate that, by 2020, 90% of those with HIV will be diagnosed, 90% of those diagnosed will be on ART, and 90% of those on ART will be virally suppressed.) Because these new HIV incidence projections affect the projected impact of VMMC, we used the new DMPPT 2 model to re-run the 2011 impact projections from the first version of DMPPT [2], allowing us to compare results between the two models. Further exploration of the impact and cost of scaling up VMMC in the context of the 90-90-90 goals is described in another paper in this collection [11].

This paper assesses the impact, cost-effectiveness, and age-specific coverage attributable to circumcisions performed through the end of 2014. It compares actual progress against the initial 80% coverage targets and presents new insights on coverage and impact by age group that may help countries develop future targets and operational plans.

## Methods

IRB clearance was not required for this study, because patient records were not collected or reviewed.

### DMPPT 2.1 model

DMPPT 2.1—the model used for this exercise—is an update of the DMPPT 2.0 that is described elsewhere in this collection [3]. DMPPT 2.1 expands the functionality of the DMPPT 2.0, which began analyses in 2013, to instead begin at the onset of each country's VMMC program. In the updated model, the user can input the number of VMMCs by age stratum for each year prior to the present and estimate the impact and age-specific MC coverage attributable to circumcisions conducted to date. The user can also set age-specific coverage targets for any future year, whereas the 2.0 version was set to reach the targets by the end of 2018. The 2.1 model allows the user to specify the average number of years from HIV infection to ART initiation; this information is then used to calculate treatment cost and cost savings. Finally, the new version of the model allows the user to specify the start and end dates of two periods over which the various model outputs are calculated (number and percentage of HIV infections averted, number of VMMCs per HIV infection averted, total VMMC program cost, cost per HIV infection averted, HIV treatment costs averted, and cost savings). Some model formulas were modified [3] to allow the model to track coverage of circumcision by age group over time with greater accuracy. These are detailed in S1 Appendix.

### Data sources

The annual numbers of VMMCs conducted for the 14 priority countries in eastern and southern Africa were reported through the joint WHO and UNAIDS Global AIDS Response Progress Reporting (GARPR) online system and direct reports from ministries of health. Data from both sources were cross-checked and verified by WHO and UNAIDS country offices and ministries of health before final publication by WHO and UNAIDS. For Kenya, national numbers from WHO were used for the figures and table reporting total numbers of circumcisions conducted in the country, whereas modeling was conducted at the provincial level only for Nyanza Province using VMMC service statistics for Nyanza province obtained from national program records. Figures and tables showing the results of the modeling analyses reflect these provincial numbers.

Calculating the number of HIV infections averted and other model outputs required the annual age disaggregation for VMMCs already performed. This information was provided from data in the 2014 Annual Performance Report (APR) of the U.S. President’s Emergency Plan for AIDS Relief (PEPFAR), or imputed if unavailable from a country or year. For Botswana, Nyanza Province (Kenya), Lesotho, Malawi, Namibia, Rwanda, Uganda, and Zambia, the annual totals through 2013 were disaggregated according to the distribution of VMMCs by age in 2014 (S1 Table). For the other countries, the age disaggregation of prior years was available or imputed through other approaches. The number of VMMCs by age group is detailed in S2–S4 Tables for Mozambique, Swaziland, and Tanzania. For South Africa, these data are available in S1 Table in [6], and for Zimbabwe, they are in a S1 Table in [12].

Baseline MC prevalence was obtained from Demographic Health Surveys (DHS) or AIDS Indicator Surveys (AIS) from the most recent year before the start of the VMMC program in each country [13–25]. In-country teams were consulted to confirm the validity of all data; in the cases of Lesotho and Malawi, the teams advised dividing baseline estimates in half in response to a study indicating that about 50% of traditional circumcisions were incomplete [26].

VMMC costs for each country (S5 Table) were estimated based on a recent facility-based costing study conducted in South Africa, which derived a unit cost of $132 per circumcision, of which $70.64 was the cost of labor [27]. The labor costs for other countries were multiplied by the ratio of each country's purchasing power parity-adjusted per capita gross national income in 2014 [28] to that of South Africa the same year. The other cost categories (consumables, continuous quality improvement, overhead, training, equipment, and vehicles) were assumed not to vary across countries. Demand creation costs were not included in the unit cost. The resulting total country VMMC unit cost was increased by a nominal 15% to account for above-facility level costs, such as management and other programming [29]. An annual discount rate of 3% starting in 2011 was applied both to costs and HIV infections averted for the calculation of costs per HIV infection averted.

### Setup of Goals files

The DMPPT 2.1 model was populated with population, mortality, and HIV incidence and prevalence projections exported from a Spectrum/Goals [30] file for each country, as previously described [3]. The Goals model within the Spectrum suite of models is a compartmental deterministic model incorporating demography, HIV epidemiology, sexual behavior, HIV disease progression, and the impact of HIV treatment and prevention interventions on mortality and new HIV infections.

Base Goals files were prepared for each country based on publicly available data from surveys such as the DHS. Historical ART and prevention of mother-to-child transmission of HIV (PMTCT) service statistics were imported from the nationally validated Spectrum/AIDS Impact Module (AIM) [31] file for each country. (UNAIDS and countries worldwide use Spectrum/AIM to estimate annual HIV prevalence and incidence based on surveillance data combined with program statistics on ART and PMTCT.) Future ART scale-up was modified from the base file to reflect the 90-90-90 treatment targets as follows: The CD4 threshold for adult treatment was set to 500 for 2015 and 2016, and to 999 starting in 2017, if not already set at those levels or higher. The age below which all HIV-positive (HIV+) children should be on treatment was set to 180 months starting in 2017. Adult and child ART coverage was scaled up by linear interpolation from the 2014 level to 81% (90% tested x 90% of those tested on ART) in 2020 and 90% (95% tested x 95% on ART) in 2030. The “90% virally suppressed” target was modeled by the “ART effect” parameter (ratio of infectiousness with ART to that without ART) interpolated linearly from the 2015 level in the base file to 0.10 in 2020 (90% virally suppressed) and 0.05 in 2030 (95% virally suppressed).

### Uncertainty analyses

The Goals uncertainty analysis/model fitting tool [32] was used to estimate uncertainty in HIV incidence projections based on the range of possible model parameters that could provide a good fit to the historical HIV prevalence data. The specific procedures for the uncertainty analysis conducted for this paper are detailed in S1 Appendix. Because MC coverage by age group is not affected by HIV incidence but rather by population projections and baseline MC coverage levels, and because the uncertainty in these inputs was not quantifiable, we did not conduct uncertainty analyses on the MC coverage estimates.

## Results

### Progress toward numerical targets

**Fig 1:**
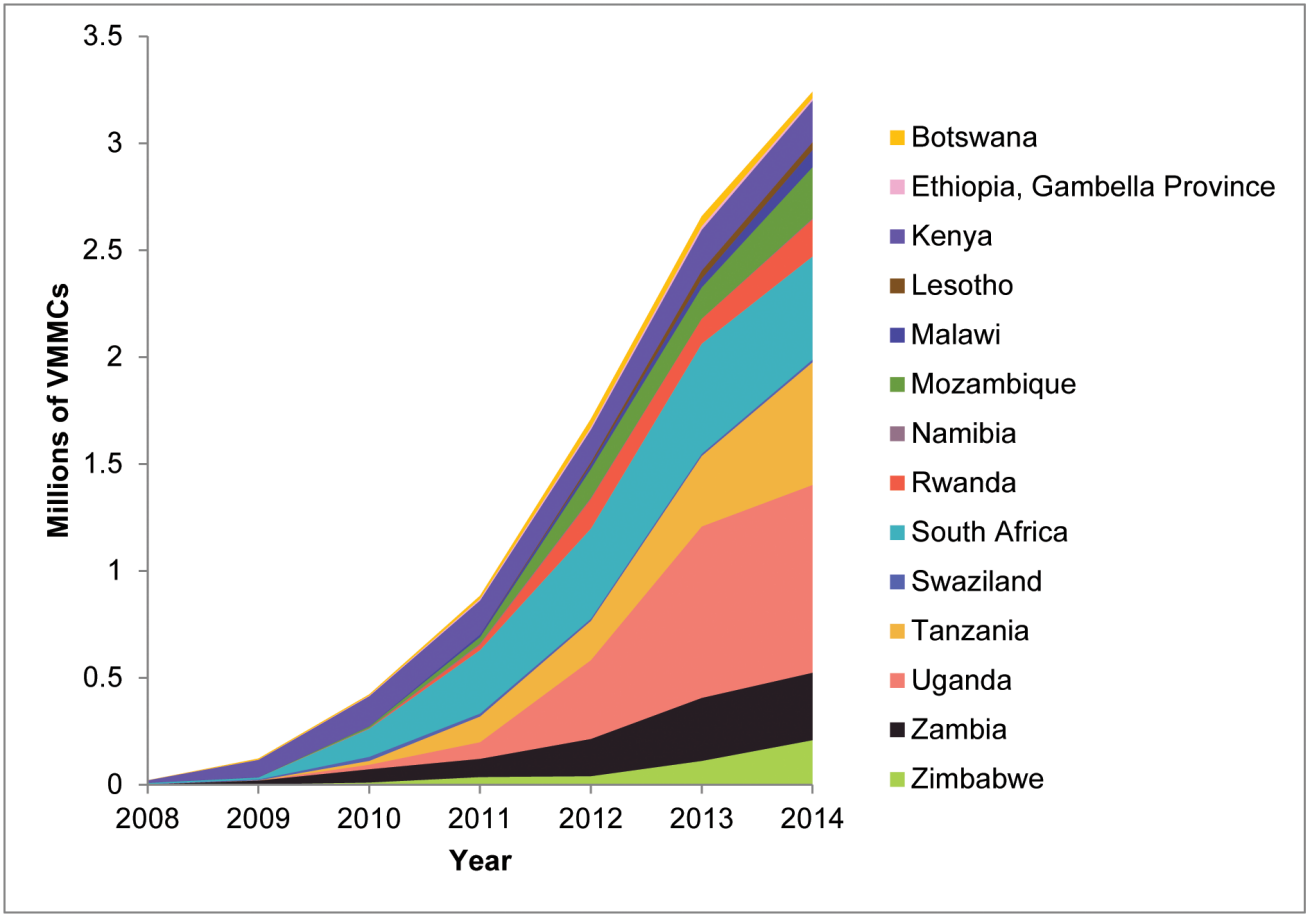
Annual numbers of VMMCs conducted in eastern and southern Africa by country, 2008–2014. Reprinted with permission from [9]: WHO progress brief, voluntary medical male circumcision for HIV prevention in 14 priority countries in East and southern Africa. Available from: http://www.who.int/hiv/topics/malecircumcision/male-circumcision-info-2014/en/.

**Fig 2:**
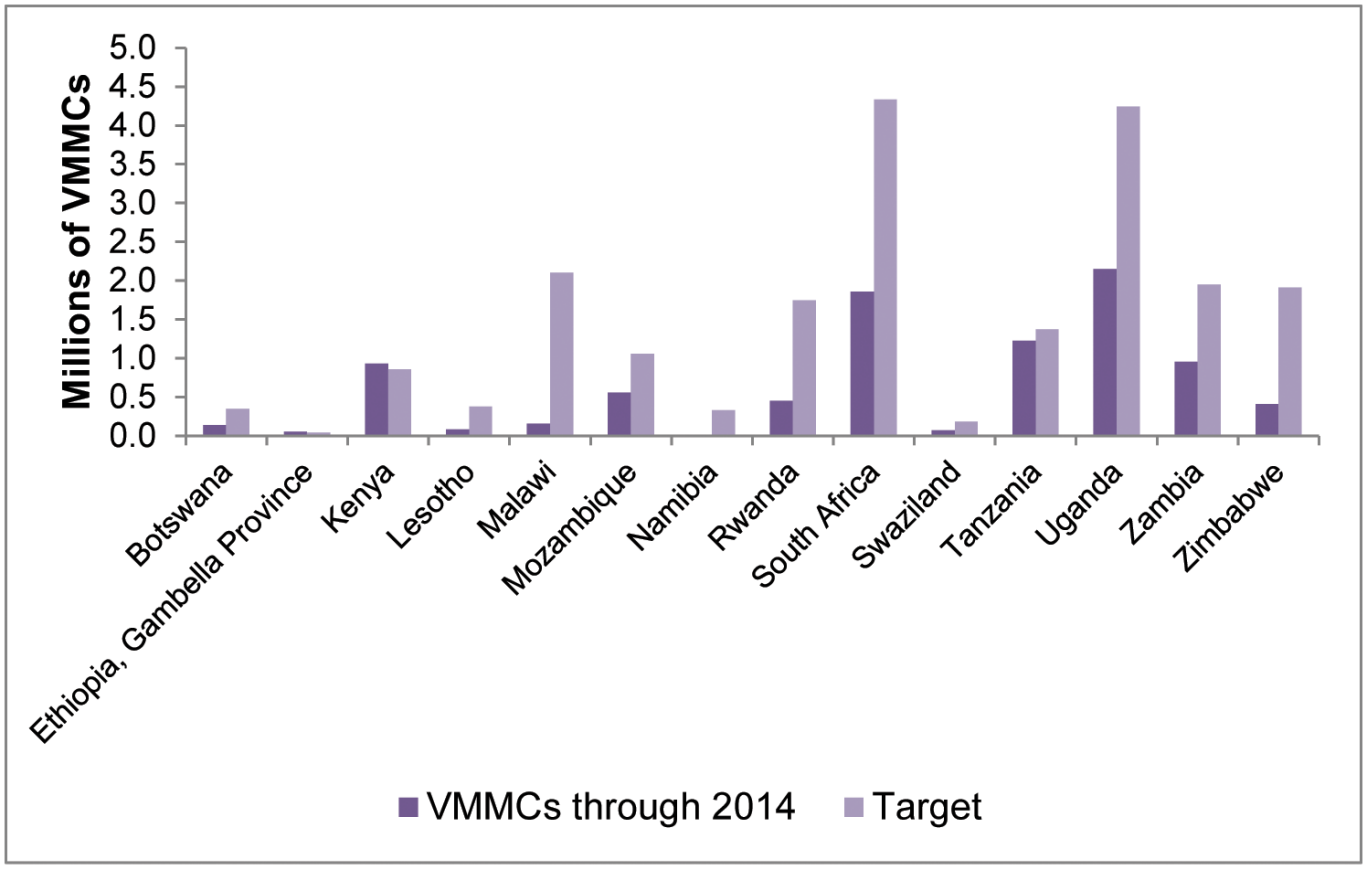
Progress toward 80% MC coverage among males ages 15–49 as of 2014, by country. This figure shows the number of VMMCs conducted through 2014 in each of the 14 priority countries in comparison with the estimated VMMC target number required to reach 80% male circumcision coverage among males ages 15–49.

The total number of VMMCs performed annually in the 14 priority countries increased from 21,000 in 2008 (programs started in only five countries) to 1.71 million in 2012 (programs started in all countries), to 2.66 million in 2013, and to 3.24 million in 2014, for a cumulative total of 9.1 million by the end of 2014 (Fig 1 and S6 Table). The greatest cumulative numbers of VMMCs were performed in Uganda (2.15 million), South Africa (1.86 million), and Tanzania (1.23 million), and the greatest annual increase in the number of VMMCs performed occurred in 2013 (0.95 million more VMMCs than in 2012). The 9.1 million represents 43% of the previously estimated 20.9 million VMMCs required to reach 80% coverage of males ages 15–49 years by the end of 2015 [2,33,34]. Kenya and Ethiopia, each with only one province with MC prevalence below 80% at baseline, have exceeded their numerical targets (Fig 2). Tanzania, where 11 of 30 regions with low MC and high HIV prevalence were prioritized, had performed almost 90% of the modeled target number. Botswana (41%), Mozambique (53%), South Africa (43%), Swaziland (38%), Uganda (51%), and Zambia (49%) are about midway toward their estimated numerical targets, while Lesotho (22%), Malawi (7%), Namibia (6%), Rwanda (26%) and Zimbabwe (22%) have yet to achieve one-quarter of the projected numbers.

### Impact of VMMCs conducted through 2014

For 12 VMMC priority countries and Nyanza Province in Kenya (no model for Gambella Province in Ethiopia is available), we used the DMPPT 2.1 model to project the impact from 2009 through 2025 of the VMMCs conducted from the beginning of the program in each country through the end of 2014. We compared that with the projected impact through 2025 of scaling up to 80% MC coverage among men ages 15–49 between 2011 and 2015 and maintaining 80% coverage thereafter, as in the first 13-country modeling analysis [2]. In this revised projection, we used updated HIV incidence projections based on new HIV surveillance data, and assumed achievement of the 90-90-90 treatment goals announced by UNAIDS in 2014 [10].

**Fig 3:**
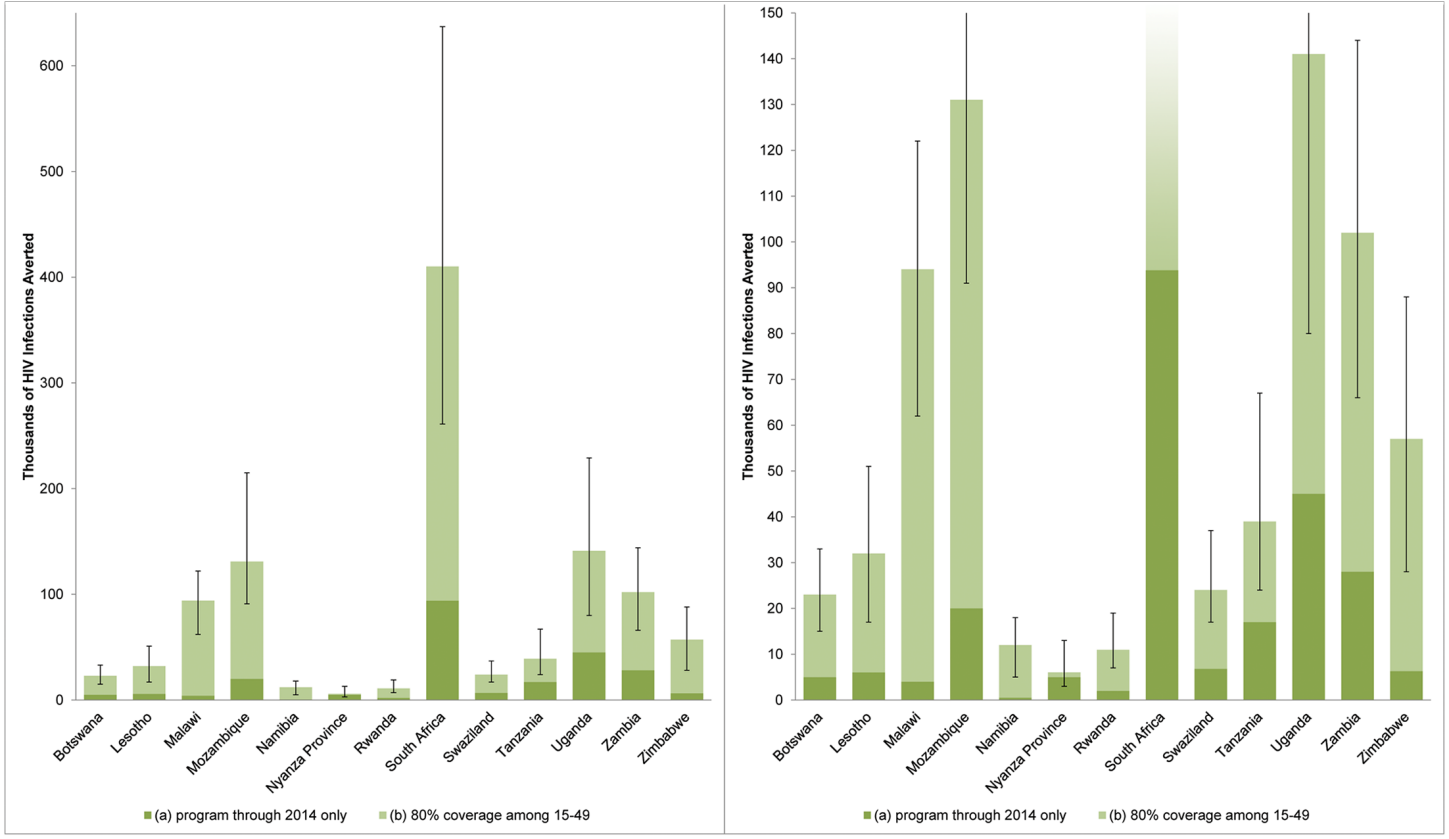
Projected HIV infections averted by 2025. (a) VMMCs performed through end 2014; (b) Scenario assuming scale-up to 80% MC coverage among males ages 15–49 by 2015 and maintained at 80% coverage through 2025. HIV infections averted are not discounted.

The results are shown in Fig 3 and detailed in S7 Table. The VMMCs conducted through 2014 in the 12 countries and Nyanza Province are projected to avert a total of 240,000 (uncertainty interval 229,000–572,000) infections through 2025, compared to 1.082 million (0.744–1.839 million) if each country had reached 80% MC coverage by the end of 2015 and maintained that level of coverage through 2025 (S7 Table). HIV infections averted are not discounted in this figure, so that numbers may be compared with results of the prior DMPPT modeling exercise [2]. South Africa (94,000), Uganda (45,000), Zambia (28,000), and Mozambique (20,000) demonstrated the highest projections for numbers of HIV infections averted through 2025. The countries with the highest ratio of HIV infections averted by VMMCs conducted through 2014 compared with those of the 80% coverage by 2015 scenario are Nyanza Province, Kenya (83%), Tanzania (44%), Uganda (32%), and Swaziland (29%).

### Cost per HIV infection averted

**Fig 4:**
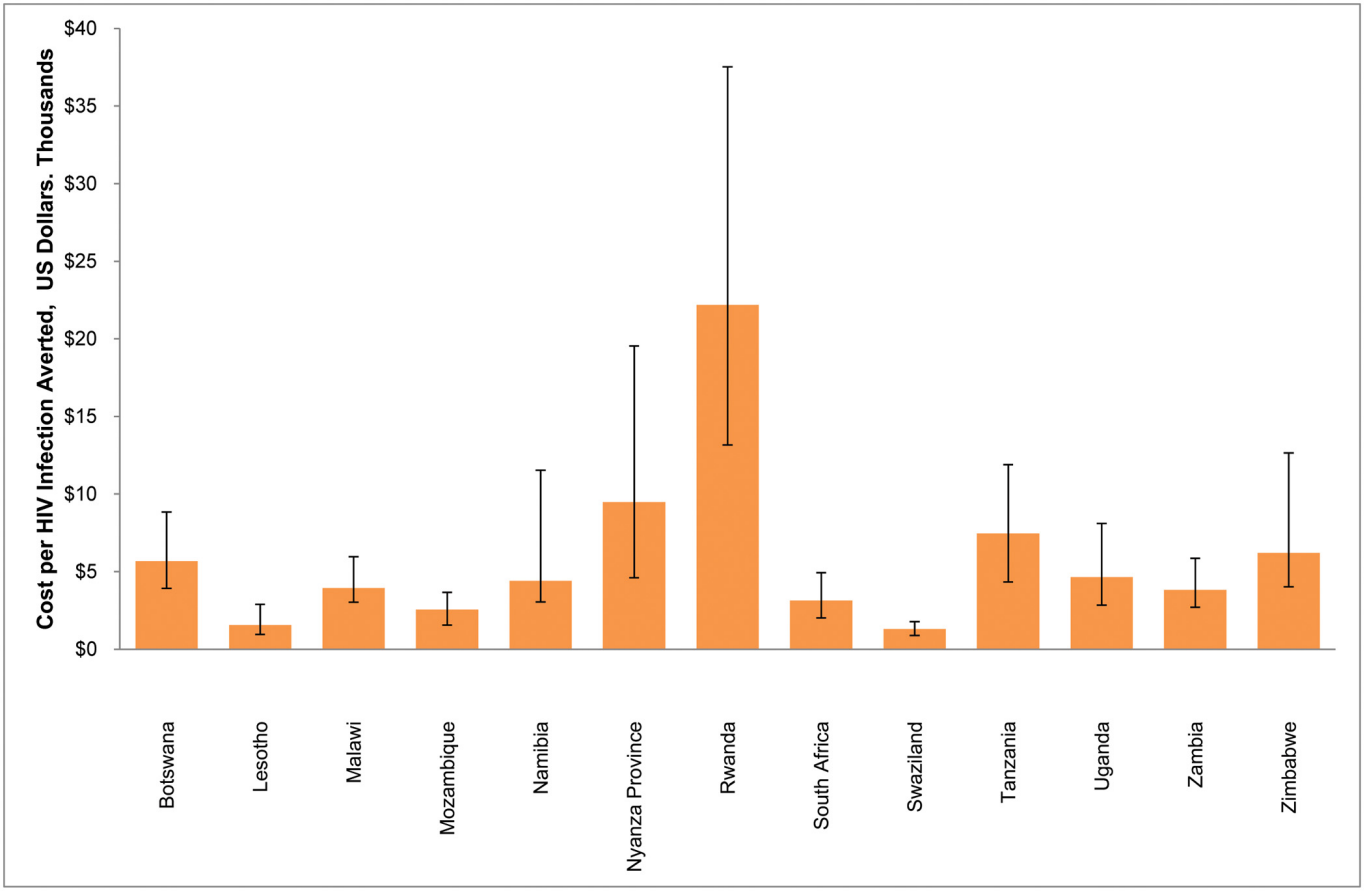
Cost per HIV infection averted, 2009–2025, for VMMC priority countries. Error bars represent 95% uncertainty bounds.

Fig 4 shows the modeled cost per HIV infection averted for VMMCs conducted through 2014, with HIV infections averted projected between 2009 and 2025, inclusive. There is a wide range of costs per HIV infection averted, from $1,300 in Swaziland to $22,200 in Rwanda, with a median of $4,400. For 10 of the 13 countries, the cost per HIV infection averted is less than $7,000. The countries with the highest cost per HIV infection averted have the lowest projected HIV incidence over the period assessed.

VMMC unit cost is a highly uncertain input parameter, so we conducted sensitivity analyses, varying the unit cost +/- 50%. In S1 Fig, we show the variation around the cost per HIV infection averted when only the unit cost is varied. There is a direct relationship between the unit cost and the cost per HIV infection averted, meaning that when the unit cost is 50% lower, the cost per HIV infection averted is 50% lower, and vice versa. S2 Fig shows how unit cost variation amplifies the effects of HIV incidence uncertainty. When the unit cost is 150% of the original unit cost and the HIV incidence reflects the high bound of the uncertainty around the incidence, the uncertainty bound around the cost per HIV infection averted is 150% of that when only the HIV incidence is varied, and vice versa.

### MC coverage by age group

We assessed estimated MC coverage among five-year age groups between ages 10–34, men ages 35–49, and the reference age groups 10–34 and 15–49 (Table 1). Overall, circumcision coverage more than doubled among men ages 15–49 years in Botswana, Rwanda, and Swaziland, and nearly doubled in Zimbabwe, with the greatest absolute and relative increase in prevalence among 15- to 19- and 20- to 24-year-olds. These four countries have also seen substantial increases in circumcision prevalence in the 10–14 year age group.

**Table 1:** Modeled Estimates of VMMC Coverage by Age Group before Start of VMMC Program (“Base”) and by the Start of 2015 (%).

Country	10–14 (%)		15–19 (%)		20–24 (%)		25–29 (%)		30–34 (%)		35–49 (%)		10–34 (%)		15–49 (%)	
	Base	2015	Base	2015	Base	2015	Base	2015	Base	2015	Base	2015	Base	2015	Base	2015
**Botswana**	4	63	6	40	10	25	13	21	16	21	15	21	10	35	11	31
**Kenya [Nyanza only]**	37	64	47	96	44	90	47	73	47	61	48	59	44	78	45	75
**Lesotho**	1	23	13	34	31	43	31	40	30	39	30	40	19	36	26	39
**Malawi**	7	11	11	16	11	15	9	13	11	13	11	13	10	14	11	14
**Mozambique**	33	45	36	48	44	52	53	57	52	59	55	62	42	51	47	56
**Namibia**	16	16	20	21	26	29	25	30	25	30	24	38	18	25	21	32
**Rwanda**	3	11	10	39	16	45	17	30	18	25	12	23	12	29	14	32
**South Africa**	22	46	26	43	42	53	44	51	52	57	54	61	36	50	45	55
**Swaziland**	4	26	4	23	7	25	8	24	10	23	15	26	6	24	8	25
**Tanzania [11 priority regions]**	20	55	40	75	49	70	41	60	46	55	43	54	37	63	44	63
**Uganda**	23	40	27	63	31	63	30	42	27	37	25	35	27	50	27	49
**Zambia**	8	27	10	36	13	36	12	29	14	25	14	22	11	31	13	29
**Zimbabwe**	4	21	5	22	8	16	11	14	11	14	11	14	7	18	9	16

Table 1 shows that Nyanza Province in Kenya is projected to have reached saturation (greater than 80% MC coverage) among males ages 15–24, with nearly a 50% coverage increase over baseline levels, and near saturation among males ages 25–29. Tanzania is close to reaching 80% coverage among males ages 15–24 in the country's 11 priority regions. In these regions, coverage is estimated to have increased by 35% among males ages 10–14 and 15–19, and about 20% among males ages 20–24 and 25–29. Also notable is Uganda, with over 60% coverage among males ages 15–24: a greater than 30% increase over baseline coverage. In Botswana, the coverage among males progressed from a baseline MC prevalence of 4% to an estimated 63% among those ages 10–14, and from 6% to 40% among those ages 15–19 by the end of 2014. MC coverage in Lesotho has increased from 1% to 23% among those ages 10–14 and from 13% to 34% among those ages 15–19. In contrast, Swaziland and Zambia have seen more uniform increases in coverage across age groups.

Overall, eight of the countries increased coverage by 20% or more among males ages 15–19; six countries achieved at least a 20% increase among males ages 10–14; and five countries achieved at least this level of increase among males ages 20–24. Only one country, Tanzania, achieved a 20% increase in coverage among males ages 25–29, and no country achieved this level of increase in coverage among men age 30 and older.

### Impact by age group

**Fig 5:**
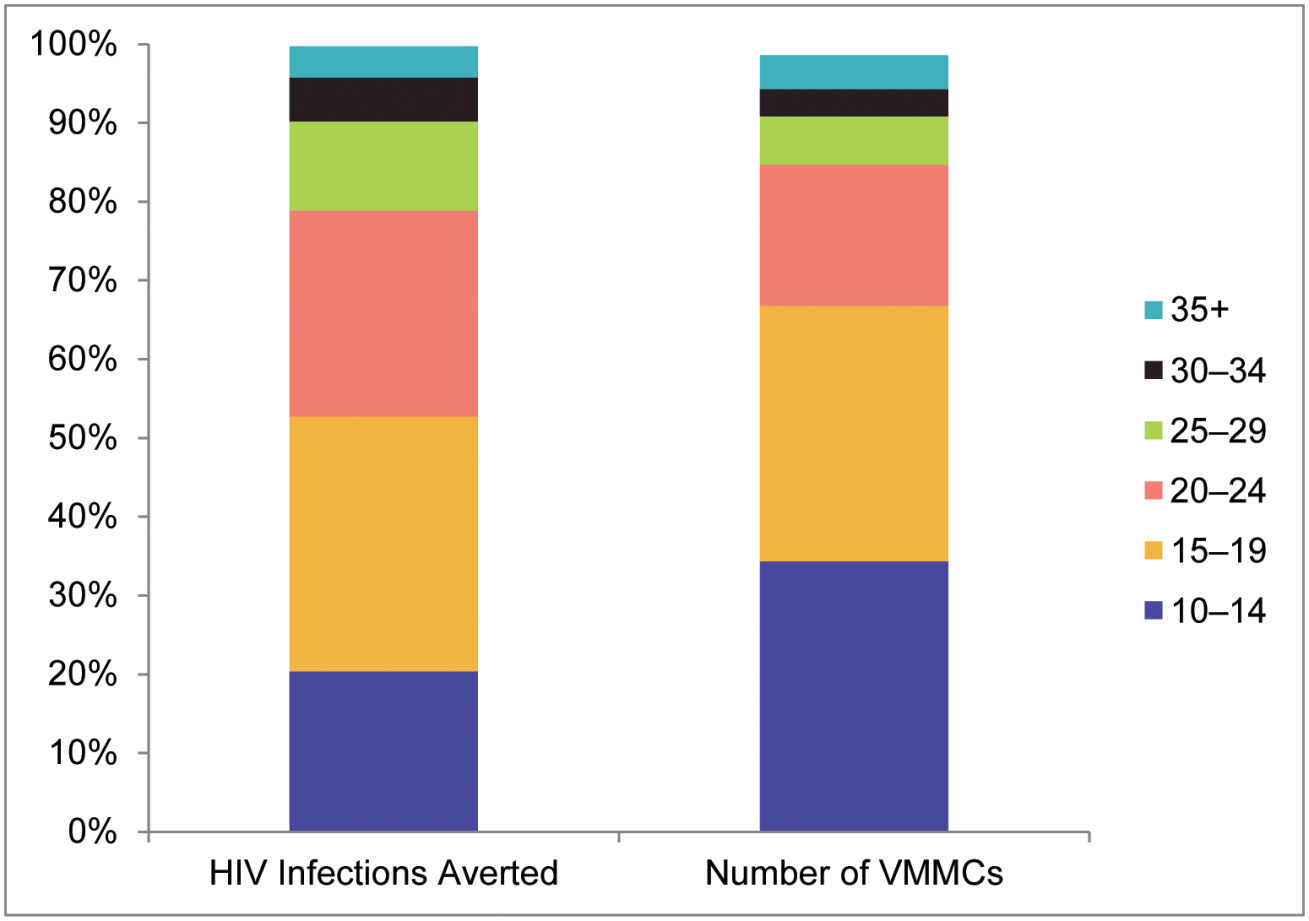
Proportion of HIV infections averted and VMMCs by age group. The left column shows the proportion of HIV infections averted attributable to VMMCs performed in each age group; the right column shows the proportion of VMMCs performed in each age group.

Given that VMMCs were not performed uniformly across age groups, that boys ages 10–14 were also included in the VMMC programs, and that the impact of circumcision differs among age groups over time [3], we compared the proportion of HIV infections averted attributable to each age group to the proportion of VMMCs performed in each age group for the 12 countries and Nyanza Province (Fig 5). Among the 240,000 projected HIV infections averted through 2025 by means of VMMCs performed through 2014 in these countries, 20% of the infections averted can be attributed to circumcisions in the age group 10–14, 32% to the age group 15–19, 26% to the age group 20–24, 11% to the age group 25–29, and 10% to age 30 and above. The proportion of HIV infections averted attributable to circumcising males ages 10–14 is smaller than the proportion of VMMC clients in this age group (34%); for ages 15–19 the two proportions are the same (32%); for ages 20–24, 25–29, and 30–34, the proportions of infections averted exceed the proportions of VMMC clients in these age groups. For clients age 35 and above, the proportions are the same (4%).

## Limitations

This study has several limitations. Age-disaggregated data for VMMCs are mostly unavailable prior to 2013, so for most countries we imputed the age-disaggregation of VMMCs based on more recent numbers. If the representation of the different age groups changed over time, our estimates of the age breakdown, and therefore our estimates of HIV infections averted, would be incorrect. In Zimbabwe [12], the representation of clients age 20 and above has decreased with time. If this is the case in other countries, the number of HIV infections averted would be slightly underestimated in our analysis. A further data limitation is that age-disaggregated data in most countries were only available from PEPFAR, so in countries where there were other VMMC implementers, we applied the age disaggregation from PEPFAR to the number of circumcisions across the country.

Additional uncertainty in the estimates comes from inherent uncertainty in modeled future HIV incidence. It is not possible to predict perfectly what course the HIV epidemic will take. The estimated decrease in HIV incidence is highly optimistic, because it assumes future reductions in HIV incidence resulting from full achievement of the 90-90-90 ART and viral suppression targets in women and men. In reality, reaching such targets is likely to be difficult, and if they are not reached, or if other factors exacerbate the HIV epidemic, the impact of scaling up VMMC would be greater than estimated here, and the cost per HIV infection averted would be lower.

Another limitation comes from the VMMC unit cost estimates. Robust cost data are not available for all countries. For the sake of internal consistency in this analysis, we estimated unit costs based on a recent costing study conducted in South Africa, with labor costs for each country adjusted based on that country’s Gross National Income. The unit cost of demand creation is difficult to determine, as it is very setting-specific, so it is not included in the cost estimates [35]. In addition, unit costs can increase or decrease over time as programs either become more efficient or expend greater resources to attract hard-to-reach clients, such as men living in remote areas or those age 20 and older. Because of these uncertainties, the cost per HIV infection averted may be either an underestimate or an overestimate for each country, and the relative cost per infection averted between countries is highly uncertain.

An important limitation in the coverage estimates comes from the assumption that the VMMC program is additive to traditional or other medical circumcisions that were implemented before the initiation of the VMMC program. The DMMPT 2.1 model can account for “replacement,” where men who would otherwise have gotten circumcised through traditional or other channels are instead circumcised through the VMMC program. However, there are no empirical data available to inform the setting of this parameter in the model. In countries where the VMMC program partially replaces other channels for circumcision, the coverage estimates reported here would be overestimates.

## Discussion

### Summary

By the end of 2014, the 14 countries scaling up VMMC had achieved 43% of the original target circumcisions needed to achieve 80% coverage among males ages 15–49, preventing a projected 240,000 HIV infections through 2025 if the 90-90-90 HIV treatment targets are achieved. The median cost was estimated at $4,400 per HIV infection averted. The proportions of VMMCs performed through 2014 compared with the targets vary across age groups and countries. Countries have generally progressed toward coverage targets faster among males ages 10–24 than those age 25 and above. Even though males ages 10–14 were not included in the original targets, they constituted nearly 35% of clients reached through 2014, and contributed to 20% of the projected HIV infections averted.

### Target achievement

Countries fall into three coverage groups with respect to target achievement: those surpassing the numbers needed (Kenya and Ethiopia); those that have reached between 50% and 75% (Tanzania, Botswana, Mozambique, South Africa, Swaziland, Uganda, and Zambia); and those with less than one-third completed (Lesotho, Malawi, Namibia, Rwanda, and Zimbabwe). Because the initial target only included males ages 15–49, the fact that more than one-third of clients were circumcised at a younger age makes interpretation of progress complex. Inclusion of clients ages 10–14 would have substantially increased the initial target. Nonetheless, because of the high acceptability of circumcision in this age group, stakeholders in several countries (e.g., Malawi, Swaziland, Tanzania, and Uganda) are now including males ages 10–14 in their targets and operational plans.

### Impact of VMMCs conducted through 2014

We reconstructed the 2011 impact projection by Njeuhmeli and colleagues using the DMPPT 2.1 model and updated HIV incidence projections based on newer HIV surveillance data and assumptions of full achievement of the 90-90-90 HIV treatment goals. With these revisions, the 80% scale-up scenario among males ages 15–49 would avert 1.1 million instead of 3.4 million HIV infections through 2025, owing to lower incidence than initially projected through 2015, as well as steep future reductions in HIV incidence anticipated from assumed near-universal access to ART and viral suppression in women and men. The implications of VMMC scale-up in the era of 90-90-90 are explored in more detail in [11].

### Cost-effectiveness

The median cost per HIV infection averted from VMMC scale-up was $4,400, and most countries had a cost per HIV infection averted of less than $7,000. This is in the same range as the cost per HIV infection averted by scaling up ART ($5,300 [36] or $8,375 [37]), Option B+ ($6,000–$23,000 [38]), or other biomedical interventions ($7,300 [39]). The other cost-effectiveness studies cited compared the cost-effectiveness of adding specific interventions before adoption of the 90-90-90 treatment goals, while we report the incremental cost-effectiveness of adding VMMC to a strategy that already includes the 90-90-90 treatment goals.

The median cost per HIV infection averted of $4,400 in this study is much higher than the initially estimated $700. The main parameter driving this difference is the lower current and projected future HIV incidence in this study, as discussed in the impact section above. Lower HIV incidence means fewer HIV infections averted, corresponding to a higher cost per HIV infection averted.

The cost per HIV infection averted is a highly uncertain parameter, because it combines the uncertainty in both the HIV incidence projections and the VMMC unit cost estimates. Sensitivity analyses were conducted to demonstrate how the VMMC unit cost uncertainty amplifies the overall uncertainty of the estimates of cost per HIV infection averted.

### Coverage by age group

An important innovation enabled by DMPPT 2.1 is the ability to estimate MC coverage and impact by age group. Results demonstrate lower coverage among older males. At the same time, VMMC programs reaching saturation in young adults or in certain regions may need to begin planning for the maintenance/sustainability phase earlier than absolute VMMC numbers might suggest. Maintaining 80% or higher coverage of VMMC over the long term involves circumcising adolescents and/or infants [40] and requires sustainable approaches to VMMC implementation [41].

### Impact by age group

Using the age-disaggregated numbers of VMMCs conducted to date, we estimated the contribution of VMMCs in each age group to the overall projected number of HIV infections averted. Circumcisions among males ages 20–34 contribute more to the proportion of HIV infections averted (43%) than their representation in the client population (28%) would indicate, suggesting consideration of better ways to attract these men to VMMC services. The demand for VMMC among males ages 10–14 has been high, and though their contribution to impact is delayed in comparison with that of adults, they were responsible for 20% of the overall projection of HIV infections averted through 2025.

### Conclusion

Remarkable progress has been made in implementing VMMC services—which reached well over 10 million men in 2015—to prevent an infection that has ravaged sub-Saharan Africa for nearly 30 years and resulted in a public health burden for individuals, families, communities, and economies. Furthermore, VMMC programs have reached a large population that generally does not access health services, providing them with a comprehensive combination prevention package that includes HIV testing services and linkage to HIV care and treatment. Eight years have passed, including about five years of practical service delivery experience in the priority countries. The modeling in this paper estimates the impact of the 9.1 million VMMCs performed through 2014 and updates important assumptions used in the model, primarily related to HIV incidence.

Since the introduction of VMMC and the initial modeling work, the annual number of new HIV infections has fallen to 1.4 million, and new research has identified additional HIV prevention strategies, such as prevention through treatment, with the potential to further reduce HIV incidence. Assuming achievement of the ambitious goals in women and men outlined by the UNAIDS 90-90-90 strategy by 2020, the projected impact of VMMC scale-up to 80% by the end of 2015 shifts from 3.4 million to a conservative estimate of 1.1 million HIV infections averted. If 90-90-90 is not met, the impact of VMMC will be even greater [11]. Even if VMMC programs had stopped circumcising at the end of 2014, the 9.1 million males circumcised are projected to contribute to almost a quarter-million HIV infections prevented through 2025, in turn averting as many as 10 million person-years of ART [42,43] and providing substantial cost savings from other comorbidities associated with HIV infection in an era of high ART coverage [44].

Findings from this assessment can inform the next UNAIDS and WHO five-year strategies and actions. In the Fast Track context, with ever-decreasing HIV incidence trend estimates, VMMC remains an impactful and cost-effective intervention to reduce HIV incidence in eastern and southern Africa. Improved age and geographic monitoring will be essential to inform program prioritization and impact estimates. Synergies to achieve the 2020 goals of HIV incidence reduction should be enhanced with tailoring by age groups. To achieve the UNAIDS goals for 2020 and 2030, all effective interventions, including VMMC, are required at scale.

## Supporting Information

S1 Appendix Supplemental Methods. (DOCX)

S1 Table Age Distribution of VMMCs Applied to Annual Reported VMMCs, by Country. Indicated percentages were multiplied by total number of VMMCs conducted in each year in Botswana, Nyanza Province (Kenya), Lesotho, Malawi, Namibia, Rwanda, Uganda, and Zambia to provide numbers of VMMCs disaggregated by age group and year—a required input for the DMPPT 2.1 model.
(DOCX)

S2 Table Number of VMMCs by Age and Year, Mozambique. Source: Mozambique Ministry of Health. VMMCs for 2010–2012 for each province were disaggregated by age based on the 2013 provincial age distribution, obtained from national program data. Data from 2013 and 2014 were already disaggregated except for ages 25–49. Because a disaggregation for this age group was unavailable for any year from Mozambique, these were disaggregated based on the age distribution of circumcisions conducted in Malawi in PEPFAR FY 2013, based on PEPFAR program data. VMMCs for ages 50+ were put into the 50–54 year age group.
(DOCX)

S3 Table Number of VMMCs by Age and Year, Swaziland. Source: Swaziland Ministry of Health.
(DOCX)

S4 Table Number of VMMCs by Age and Year, Tanzania. Source: Tanzania Ministry of Health. VMMCs for males ages 25–49 for each year of the program were disaggregated based on the age distribution of circumcisions reported from Jhpiego in 2013.
(DOCX)

S5 Table VMMC Unit Costs, by Country. See Methods section for description of sources.
(DOCX)

S6 Table Progress toward the Numerical Targets Required to Reach 80% Male Circumcision (MC) Coverage among Males Ages 15–49, According to Modeling Conducted in Njeuhmeli, et al, 2011 [2]. Targets for Ethiopia and Kenya were adopted later and published in [33]. Source: [33].
(DOCX)

S7 Table Projected Number of HIV Infections Averted by 2025 by VMMCs Performed through End 2014, and Projected Number Averted by 80% Coverage among Males Ages 15–49 by 2015 and Maintained at 80% Coverage Through 2025. Numbers in parentheses represent 95% uncertainty bounds. See text for description of methods.
(DOCX)

S1 Fig Sensitivity analysis 1: Cost per HIV infection averted with unit cost varied. Error bars represent the cost per HIV infection averted with the unit cost varied by +/- 50%.
(TIF)

S2 Fig Sensitivity analysis 2: Cost per HIV infection averted with unit cost varied on top of HIV incidence uncertainty. Lower error bars for each country are derived from a model employing the lower uncertainty bound for HIV incidence and the unit cost multiplied by 50%. Upper error bars are derived from a model employing the upper uncertainty bound for HIV incidence and the unit cost multiplied by 150%. HIV incidence uncertainty methods are described in S1 Appendix.
(TIF)
