# Correction: Assessing Progress, Impact, and Next Steps in Rolling Out Voluntary Medical Male Circumcision for HIV Prevention in 14 Priority Countries in Eastern and Southern Africa through 2014

**DOI:** 10.1371/journal.pone.0163757

**Published:** 2016-09-22

**Authors:** Katharine Kripke, Emmanuel Njeuhmeli, Julia Samuelson, Melissa Schnure, Buhle Ncube, Shona Dalal, Timothy Farley, Catherine Hankins, Anne G. Thomas, Jason Reed, Peter Stegman, Naomi Bock

Dr. Buhle Ncube should be included in the author byline as the fifth author. Dr. Ncube’s affiliation is the World Health Organization, Harare, Zimbabwe. The contributions of this author are: Conceived and designed the experiments, Performed the experiments, Analyzed the data, Contributed reagents/materials/analysis tools, Wrote the paper, Other: contributed to investigation and resources.

The correct citation is: Kripke K, Njeuhmeli E, Samuelson J, Schnure M, Ncube B, Dalal S, et al. (2016) Assessing Progress, Impact, and Next Steps in Rolling Out Voluntary Medical Male Circumcision for HIV Prevention in 14 Priority Countries in Eastern and Southern Africa through 2014. PLoS ONE 11(7): e0158767. doi:10.1371/journal.pone.0158767
