# Correction: Assessing Progress, Impact, and Next Steps in Rolling Out Voluntary Medical Male Circumcision for HIV Prevention in 14 Priority Countries in Eastern and Southern Africa through 2014

**DOI:** 10.1371/journal.pone.0169698

**Published:** 2017-01-03

**Authors:** Katharine Kripke, Emmanuel Njeuhmeli, Julia Samuelson, Melissa Schnure, Shona Dalal, Timothy Farley, Catherine Hankins, Anne G. Thomas, Jason Reed, Peter Stegman, Naomi Bock

There is an error in the Funding statement. The number for the Cooperative Agreement Project SOAR (Supporting Operational AIDS Research) is incorrect. The correct number is OAA-A-14-00060.
