# Supplementary material for: Assessing Progress, Impact, and Next Steps in Rolling Out Voluntary Medical Male Circumcision for HIV Prevention in 14 Priority Countries in Eastern and Southern Africa through 2014
Source: PLoS One. 2016 Jul 21;11(7):e0158767. doi: 10.1371/journal.pone.0158767 (PMC4955652; doi:10.1371/journal.pone.0158767)
Supplement: S1 Appendix — (DOCX) [file pone.0158767.s001.docx]

## Supplemental Methods for VMMC progress manuscript

### Differences in calculations between DMPPT 2.0 and DMPPT 2.1:

The DMPPT 2 model tracks the number of males circumcised by the VMMC program separately from those circumcised through traditional or other channels (collectively termed “baseline” circumcisions). In version DMPPT 2.0, the number of circumcised males belonging to five-year age group *a* in year *t* for both program and baseline circumcisions was given by

$$M_{c,a,t}=M_{c,a,t-1}*(1-\mu_{a,t-1})+(NewM_{c,a,t-1}+AgingIn_{a,t-1}-AgingOut_{a,t-1})*\left(1-\frac{\mu_{a,t-1}}{2}\right)$$

The number circumcised at the beginning of year *t* was the number at the beginning of the previous year *t-1*, plus new circumcisions performed during year *t-1* and net circumcisions aging into and out of group *a*, all adjusted for mortality. Existing circumcisions in the age group were subject to a full year of mortality, so *M_c,a,t-1_* was multiplied by (1 – *μ_a,t-1_*). New circumcisions and circumcisions aging in and out spend only half a year in group *a* on average, and were therefore subject to only half a year’s mortality, (1 – *μ_a,t-1_*/2).

And the number of new program and baseline circumcisions performed each year was given by

$$NewM_{c,a,t}=P_{a,t}*C_{a,t}-(M_{c,a,t}+AgingIn_{a,t}+AgingOut_{a,t})$$

The number of new circumcisions in age group *a* that need to be conducted in year *t* (*New M_c,a,t_*) was the number of males in the population in age group *a* (*P_a,t_*), multiplied by the desired coverage for that age group and year (*C_a,t_*), minus the number of males already circumcised (*M_c,a,t_*), plus the number of circumcised males aging in, minus those aging out.

In version DMPPT 2.1, we introduced a new parameter, *Rep*, which controls the degree to which program circumcisions replace baseline circumcisions. In addition to addressing programmatic realities, this was needed in order to address some anomalies in the coverage estimates that occurred under certain conditions. *Rep* represents the degree to which VMMC program circumcisions draw from populations that would have circumcised through traditional or other channels in the absence of the VMMC program. When *Rep* = 0, all program circumcisions are drawn from the population of males who would not be circumcised through baseline channels. When *Rep* = 1, program circumcisions are first performed among men who would have been circumcised through baseline channels in the absence of the program. *Rep* is taken to be the same for all age groups and constant over time, and so can be interpreted as the average replacement fraction over age groups and time.

With the addition of *Rep*, new circumcisions performed by the VMMC program and through baseline channels are calculated differently. The numbers of new circumcisions performed by the VMMC program and through baseline channels are given by the following equations, where *P_a,t_* is the total number of males (circumcised and uncircumcised) in age group *a* in year *t* and *C_a,t_* is the coverage target in year *t*. In the baseline scenario, coverage is assumed to be constant at the baseline, pre-VMMC program rate of *C_a,baseline_* for all years.

$$NewM_{p,a,t}=\frac{P_{a,t+1}*C_{a,t+1}-\left[M_{c,a,t}*(1-\mu_{a,t})+(AgingIn_{p,a,t}-AgingOut_{p,a,t})*\left(1-\frac{\mu_{a,t}}{2}\right)\right]}{\left(1-\frac{\mu_{a,t}}{2}\right)}$$

$$NewM_{b,a,t}=\frac{P_{a,t+1}*C_{a,baseline}-\left[M_{b,a,t-1}*(1-\mu_{a,t})+(AgingIn_{b,a,t}-AgingOut_{b,a,t})*\left(1-\frac{\mu_{a,t}}{2}\right)\right]}{\left(1-\frac{\mu_{a,t}}{2}\right)}-NewM_{p,a,t}*Rep$$

The number of new program circumcisions to be performed in year *t* is expressed as the difference between the target number needed at the beginning of year *t+1* and the number of already-circumcised men remaining in group *a* at the end of year *t*. As before, *M_c,a,t-1_* is subject to a full year of mortality, whereas circumcisions aging in and aging out are subject to only a half year of mortality on average. This quantity must then be inflated to allow for mortality among newly-circumcised males during year *t*, again half a year on average. The equation for new baseline circumcisions is the same, with two exceptions: 1) we subtract the fraction of program circumcisions which replace baseline circumcisions (*NewM_p,a,t_* * *Rep*), and 2) we use the number of baseline circumcisions (*M_b,a,t_*) in place of the total number of circumcisions (*M_c,a,t_*). The latter is because we assume that the number or coverage of baseline circumcisions remains constant at pre-VMMC program levels, and does not depend on the number or coverage of program circumcisions, except through the replacement term.

### Uncertainty analyses

The Goals uncertainty analysis/ model fitting tool [[1](#_ENREF_1)] was used to estimate the uncertainty in HIV incidence projections based on the range of possible model parameters that could provide a good fit to the historical HIV prevalence data. All parameters in the tool were varied, using the default ranges for variation of each parameter, except those that related to male circumcision, which remained fixed. The uncertainty analysis was run with 1000 random draws. The historical fit file from this run was loaded into the “future uncertainty” projection part of the tool. The top 20 sets of parameters were used with 100 random draws to generate a set of 100 possible HIV incidence curves.

The lower uncertainty bounds for each output (number of HIV infections averted, cost per HIV infection averted) were generated from a DMPPT 2.1 file in which the age-specific HIV incidence for each year of the model was multiplied by the 2.5^th^ percentile of the 100 HIV incidence curves for that year divided by the incidence from the parent 90-90-90 Goals file for that year. Likewise the upper uncertainty bound was based on a DMPPT 2.1 file in which the HIV incidence was scaled by the 97.5^th^ percentile of the 100 HIV incidence curves generated by the uncertainty tool. The reported number of HIV infections averted and cost per HIV infection averted are based on a DMPPT 2.1 file in which the annual age-specific HIV incidence was scaled by the median incidence from the 100 incidence curves generated by the uncertainty tool.

## References

REFERENCES

1. (2011) Goals Manual: A Model for Estimating the Effects of Interventions and Resource Allocation on HIV Infections and Deaths. Glastonbury, CT USA.
