# Supplementary material for: Assessing Progress, Impact, and Next Steps in Rolling Out Voluntary Medical Male Circumcision for HIV Prevention in 14 Priority Countries in Eastern and Southern Africa through 2014
Source: PLoS One. 2016 Jul 21;11(7):e0158767. doi: 10.1371/journal.pone.0158767 (PMC4955652; doi:10.1371/journal.pone.0158767)
Supplement: S1 Table — Indicated percentages were multiplied by total number of VMMCs conducted in each year in Botswana, Nyanza Province (Kenya), Lesotho, Malawi, Namibia, Rwanda, Uganda, and Zambia to provide numbers of VMMCs disaggregated by age group and year—a required input for the DMPPT 2.1 model. (DOCX) [file pone.0158767.s002.docx]

Supplemental Table 1: Age distribution of VMMCs applied to annual reported VMMCs by country, to provide model inputs.

|  | **<1 (%)** | **1–*9 (%)*** | **10–14 (%)** | **15–19 (%)** | **20–24 (%)** | **25–49 (%)** | **50+ (%)** |
| --- | --- | --- | --- | --- | --- | --- | --- |
| **Botswana** | 0 | 9 | 60 | 16 | 7 | 9 | 1 |
| **Kenya** | 0 | 4 | 42 | 32 | 11 | 11 | 0 |
| **Lesotho** | 0 | 0 | 37 | 28 | 14 | 19 | 1 |
| **Malawi** | 0 | 1 | 33 | 36 | 16 | 14 | 0 |
| **Namibia** | 0 | 0 | 10 | 19 | 24 | 44 | 2 |
| **Rwanda** | 0 | 0 | 13 | 39 | 30 | 18 | 0 |
| **Uganda** | 0 | 0 | 25 | 40 | 25 | 7 | 2 |
| **Zambia** | 0 | 6 | 30 | 27 | 18 | 18 | 1 |
| **Total** | 0 | 1 | 35 | 33 | 17 | 13 | 1 |

Source: PEPFAR 2014 annual program reporting data
