# Supplementary material for: Assessing Progress, Impact, and Next Steps in Rolling Out Voluntary Medical Male Circumcision for HIV Prevention in 14 Priority Countries in Eastern and Southern Africa through 2014
Source: PLoS One. 2016 Jul 21;11(7):e0158767. doi: 10.1371/journal.pone.0158767 (PMC4955652; doi:10.1371/journal.pone.0158767)
Supplement: S2 Table — Source: Mozambique Ministry of Health. VMMCs for 2010–2012 for each province were disaggregated by age based on the 2013 provincial age distribution, obtained from national program data. Data from 2013 and 2014 were already disaggregated except for ages 25–49. Because a disaggregation for this age group was unavailable for any year from Mozambique, these were disaggregated based on the age distribution of circumcisions conducted in Malawi in PEPFAR FY 2013, based on PEPFAR program data. VMMCs for ages 50+ were put into the 50–54 year age group. (DOCX) [file pone.0158767.s003.docx]

Supplemental Table 2: Number of VMMCs by age and year, Mozambique.

| **Year** | **1–9** | **10–14** | **15–19** | **20–24** | **25–29** | **30–34** | **35–39** | **40–44** | **45–49** | **>50** | **Total** |
| --- | --- | --- | --- | --- | --- | --- | --- | --- | --- | --- | --- |
| **2010** | 0 | 229 | 545 | 150 | 48 | 28 | 16 | 7 | 3 | 5 | 1,031 |
| **2011** | 7 | 6,543 | 6,771 | 3,123 | 1,142 | 656 | 371 | 170 | 70 | 93 | 18,946 |
| **2012** | 10 | 38,298 | 22,655 | 10,091 | 3,630 | 2,085 | 1,178 | 539 | 222 | 293 | 79,001 |
| **2013** | 2 | 63,166 | 39,064 | 16,012 | 5,191 | 2,982 | 1,685 | 771 | 318 | 390 | 129,581 |
| **2014** | 0 | 84,181 | 44,326 | 18,550 | 6,288 | 3,613 | 2,041 | 934 | 385 | 342 | 160,660 |

Source: Mozambique national program data

VMMCs for 2010–2012 for each province were disaggregated by age based on the 2013 provincial age distribution, obtained from national program data. VMMCs for ages 25-49 were disaggregated based on the age distribution of circumcisions conducted in Malawi in PEPFAR FY 2013, based on PEPFAR program data. VMMCs for ages 50+ were put into the 50–54 year age group.
