# Supplementary material for: Assessing Progress, Impact, and Next Steps in Rolling Out Voluntary Medical Male Circumcision for HIV Prevention in 14 Priority Countries in Eastern and Southern Africa through 2014
Source: PLoS One. 2016 Jul 21;11(7):e0158767. doi: 10.1371/journal.pone.0158767 (PMC4955652; doi:10.1371/journal.pone.0158767)
Supplement: S3 Table — Source: Swaziland Ministry of Health. (DOCX) [file pone.0158767.s004.docx]

Supplemental Table 3: Number of VMMCs by age and year, Swaziland.

| **Year** | **EIMC** | **1–9** | **10–14** | **15–19** | **20–24** | **25–29** | **30–34** | **35–39** | **40–44** | **45–49** | **>50** | **Total** |
| --- | --- | --- | --- | --- | --- | --- | --- | --- | --- | --- | --- | --- |
| **2008** |  | 1 | 1 | 84 | 278 | 348 | 240 | 101 | 41 | 16 | 18 | 1,128 |
| **2009** |  | 2 | 2 | 195 | 648 | 812 | 559 | 235 | 95 | 38 | 41 | 2,627 |
| **2010** | 155 | 80 | 433 | 4,825 | 7,698 | 3,489 | 1,933 | 850 | 438 | 236 | 263 | 20,400 |
| **2011** | 932 | 203 | 2,723 | 4,644 | 2,912 | 1,786 | 853 | 431 | 225 | 130 | 145 | 14,984 |
| **2012** | 1,155 | 221 | 5,547 | 3,276 | 1,582 | 862 | 420 | 214 | 124 | 73 | 86 | 13,560 |
| **2013** | 1,431 | 66 | 5,284 | 1,510 | 545 | 357 | 203 | 111 | 33 | 22 | 34 | 9,596 |
| **2014** | 1,153 | 847 | 6,694 | 2,031 | 670 | 290 | 195 | 108 | 47 | 29 | 56 | 12,120 |

Source: Swaziland national program data
