# Supplementary material for: Assessing Progress, Impact, and Next Steps in Rolling Out Voluntary Medical Male Circumcision for HIV Prevention in 14 Priority Countries in Eastern and Southern Africa through 2014
Source: PLoS One. 2016 Jul 21;11(7):e0158767. doi: 10.1371/journal.pone.0158767 (PMC4955652; doi:10.1371/journal.pone.0158767)
Supplement: S4 Table — Source: Tanzania Ministry of Health. VMMCs for males ages 25–49 for each year of the program were disaggregated based on the age distribution of circumcisions reported from Jhpiego in 2013. (DOCX) [file pone.0158767.s005.docx]

Supplemental Table 4: Number of VMMCs by age and year, Tanzania.

| **Year** | **1–9** | **10–14** | **15–19** | **20–24** | **25–29** | **30–34** | **35–39** | **40–44** | **45–49** | **>50** | **Total** |
| --- | --- | --- | --- | --- | --- | --- | --- | --- | --- | --- | --- |
| **2010** | 406 | 4,642 | 10,703 | 3,421 | 1,040 | 623 | 343 | 184 | 95 | 100 | 21,557 |
| **2011** | 3,401 | 43,198 | 29,871 | 31,721 | 4,060 | 2,432 | 1,338 | 718 | 373 | 239 | 117,351 |
| **2012** | 5,658 | 71,244 | 35,995 | 28,704 | 4,474 | 2,680 | 1,474 | 791 | 411 | 428 | 151,859 |
| **2013** | 2,723 | 175,889 | 119,040 | 44,257 | 17,004 | 10,186 | 5,601 | 3,007 | 1,561 | 2,111 | 381,379 |
| **2014** | 156 | 254,374 | 164,024 | 69,183 | 28,327 | 16,969 | 9,331 | 5,009 | 2,599 | 3,415 | 553,387 |

Source: Tanzania national program data
