# Supplementary material for: Assessing Progress, Impact, and Next Steps in Rolling Out Voluntary Medical Male Circumcision for HIV Prevention in 14 Priority Countries in Eastern and Southern Africa through 2014
Source: PLoS One. 2016 Jul 21;11(7):e0158767. doi: 10.1371/journal.pone.0158767 (PMC4955652; doi:10.1371/journal.pone.0158767)
Supplement: S5 Table — See Methods section for description of sources. (DOCX) [file pone.0158767.s006.docx]

Supplemental Table 5: VMMC Unit costs by country.

| **Country** | **VMMC Unit Cost ($)** |
| --- | --- |
| **Botswana** | 173 |
| **Kenya** | 89 |
| **Lesotho** | 91 |
| **Malawi** | 76 |
| **Mozambique** | 78 |
| **Namibia** | 132 |
| **Rwanda** | 81 |
| **South Africa** | 152 |
| **Swaziland** | 108 |
| **Tanzania** | 87 |
| **Uganda** | 82 |
| **Zambia** | 94 |
| **Zimbabwe** | 81 |
