# Supplementary material for: Assessing Progress, Impact, and Next Steps in Rolling Out Voluntary Medical Male Circumcision for HIV Prevention in 14 Priority Countries in Eastern and Southern Africa through 2014
Source: PLoS One. 2016 Jul 21;11(7):e0158767. doi: 10.1371/journal.pone.0158767 (PMC4955652; doi:10.1371/journal.pone.0158767)
Supplement: S6 Table — Targets for Ethiopia and Kenya were adopted later and published in [33]. Source: [33]. (DOCX) [file pone.0158767.s007.docx]

Supplemental Table 6: Progress toward the numerical targets required to reach 80% male circumcision (MC) coverage among males ages 15–49, according to modeling conducted in Njeuhmeli, et al, 2011 [[2](#_ENREF_2)]. Targets for Ethiopia and Kenya were adopted later and published in [[32](#_ENREF_32)].

| **Country** | **2008** | **2009** | **2010** | **2011** | **2012** | **2013** | **2014** | **Total** | **Numerical target (millions of VMMCs)** | **% progress toward target** |
| --- | --- | --- | --- | --- | --- | --- | --- | --- | --- | --- |
| **Botswana** | 0 | 5,424 | 5,773 | 14,661 | 38,005 | 46,793 | 30,033 | 140,689 | 0.35 | 41% |
| **Ethiopia, Gambella Province** | 0 | 769 | 2,689 | 7,542 | 11,961 | 16,393 | 11,831 | 51,185 | 0.04 | 128% |
| **Kenya** | 11,663 | 80,719 | 139,905 | 159,196 | 151,517 | 190,580 | 193,576 | 927,156 | 0.86 | 108% |
| **Lesotho** | 0 | 0 | 0 | 0 | 10,835 | 37,655 | 36,245 | 84,735 | 0.38 | 22% |
| **Malawi** | 589 | 1,234 | 1,296 | 11,881 | 21,250 | 40,835 | 80,419 | 157,504 | 2.10 | 7% |
| **Mozambique** | 0 | 100 | 7,633 | 29,592 | 135,000 | 146,046 | 240,507 | 558,878 | 1.06 | 53% |
| **Namibia** | 0 | 224 | 1,763 | 6,123 | 4,863 | 1,182 | 4,165 | 18,320 | 0.33 | 6% |
| **Rwanda** | 0 | 0 | 1,694 | 25,000 | 138,711 | 116,029 | 173,191 | 454,625 | 1.75 | 26% |
| **South Africa** | 5,190 | 9,168 | 131,117 | 296,726 | 422,009 | 514,991 | 482,474 | 1,861,675 | 4.33 | 43% |
| **Swaziland** | 1,110 | 4,336 | 18,869 | 13,791 | 9,977 | 10,105 | 12,289 | 70,477 | 0.18 | 38% |
| **Tanzania** | 0 | 1,033 | 18,026 | 120,261 | 183,480 | 329,729 | 573,845 | 1,226,374 | 1.37 | 89% |
| **Uganda** | 0 | 0 | 21,072 | 77,756 | 368,490 | 801,678 | 878,109 | 2,147,105 | 4.25 | 51% |
| **Zambia** | 2,758 | 17,180 | 61,911 | 85,151 | 173,992 | 294,466 | 315,168 | 950,626 | 1.95 | 49% |
| **Zimbabwe** | 0 | 2,801 | 11,176 | 36,603 | 40,755 | 112,084 | 209,125 | 412,544 | 1.91 | 22% |
| **Total** | 21,310 | 122,988 | 422,924 | 884,283 | 1,710,845 | 2,658,566 | 3,240,977 | 9,061,893 | 20.86 | 43% |
