# Supplementary material for: Assessing Progress, Impact, and Next Steps in Rolling Out Voluntary Medical Male Circumcision for HIV Prevention in 14 Priority Countries in Eastern and Southern Africa through 2014
Source: PLoS One. 2016 Jul 21;11(7):e0158767. doi: 10.1371/journal.pone.0158767 (PMC4955652; doi:10.1371/journal.pone.0158767)
Supplement: S7 Table — Numbers in parentheses represent 95% uncertainty bounds. See text for description of methods. (DOCX) [file pone.0158767.s008.docx]

Supplemental Table 7: Projected number of HIV infections averted by 2025 by VMMCs performed through end 2014, and projected number averted by 80% coverage among males ages 15-49 by 2015 and maintained at 80% coverage through 2025. Numbers in parentheses represent 95% uncertainty bounds.

| **Country** | **VMMC program to date** | **Scale Up to 80% MC prevalence among men ages 15–49** | **Program to date / 80% by 2015** |
| --- | --- | --- | --- |
| **Botswana** | 5,000 (3,000 , 7,000) | 23,000 (15,000 , 33,000) | 22% |
| **Kenya  [Nyanza only]** | 5,000 (3,000 , 11,000) | 6,000 (3,000 , 13,000) | 83% |
| **Lesotho** | 6,000 (3,000 , 10,000) | 32,000 (17,000 , 51,000) | 19% |
| **Malawi** | 4,000 (2,000 , 5,000) | 94,000 (62,000 , 122,000) | 4% |
| **Mozambique** | 20,000 (14,000 , 32,000) | 131,000 (91,000 , 215,000) | 15% |
| **Namibia** | 1,000 (200 , 1,000) | 12,000 (5,000 , 18,000) | 8% |
| **Rwanda** | 2,000 (1,000 , 4,000) | 11,000 (7,000 , 19,000) | 18% |
| **South Africa** | 94,000 (60,000 , 146,000) | 410,000 (261,000 , 637,000) | 23% |
| **Swaziland** | 7,000 (5,000 , 10,000) | 24,000 (17,000 , 37,000) | 29% |
| **Tanzania** | 17,000 (11,000 , 29,000) | 39,000 (24,000 , 67,000) | 44% |
| **Uganda** | 45,000 (26,000 , 74,000) | 141,000 (80,000 , 229,000) | 32% |
| **Zambia** | 28,000 (18,000 , 39,000) | 102,000 (66,000 , 144,000) | 27% |
| **Zimbabwe** | 6,000 (3,000 , 10,000) | 57,000 (28,000 , 88,000) | 11% |
| **Total** | 240,000 (229,000 , 572,000) | 1,082,000 (744,000 , 1,839,000) | 22% |
