# Supplementary figures and images for: Assessing Progress, Impact, and Next Steps in Rolling Out Voluntary Medical Male Circumcision for HIV Prevention in 14 Priority Countries in Eastern and Southern Africa through 2014
Source: PLoS One. 2016 Jul 21;11(7):e0158767. doi: 10.1371/journal.pone.0158767 (PMC4955652; doi:10.1371/journal.pone.0158767)

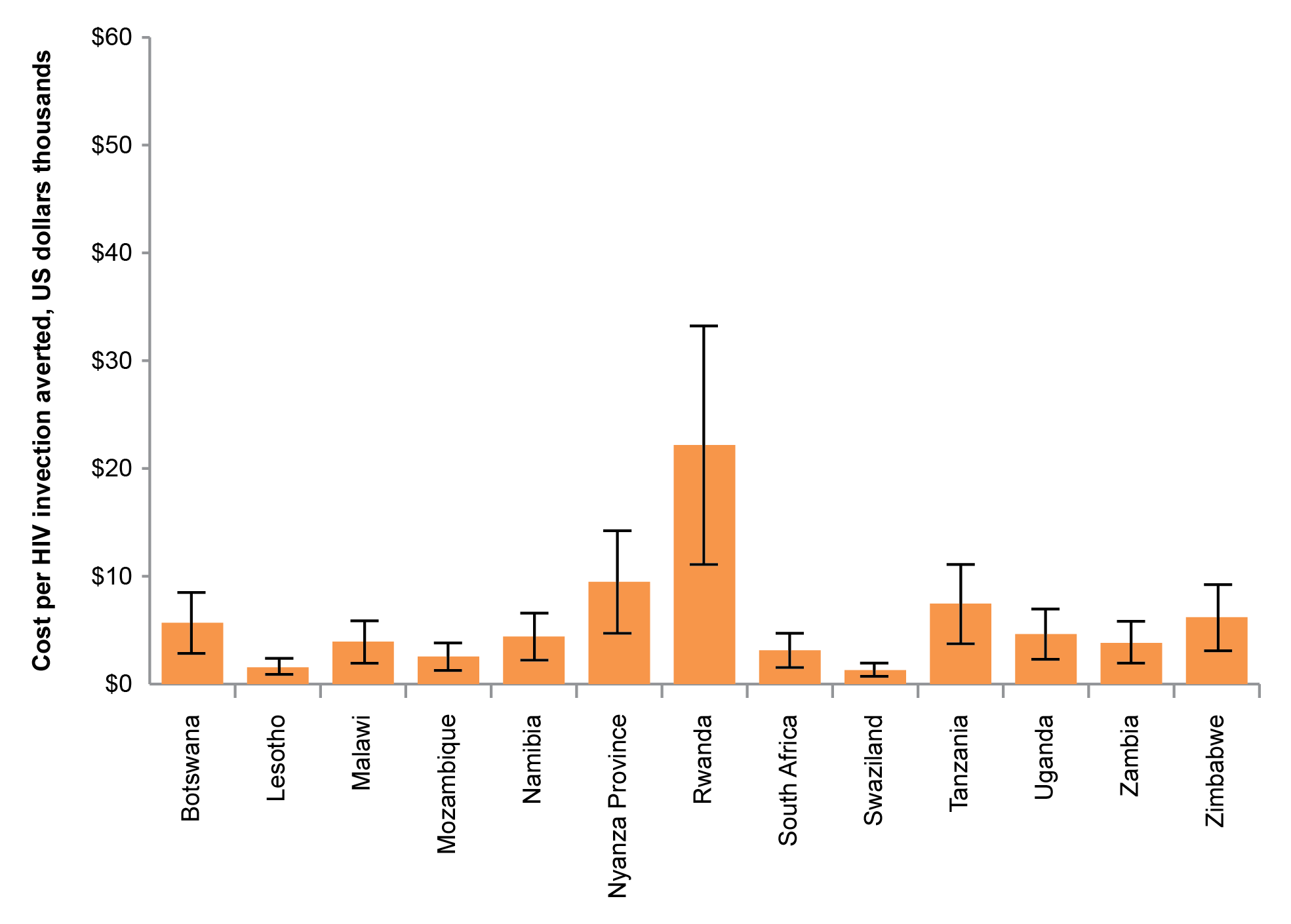

Supplement: S1 Fig — Error bars represent the cost per HIV infection averted with the unit cost varied by +/- 50%. (TIF) [file pone.0158767.s009.tif]

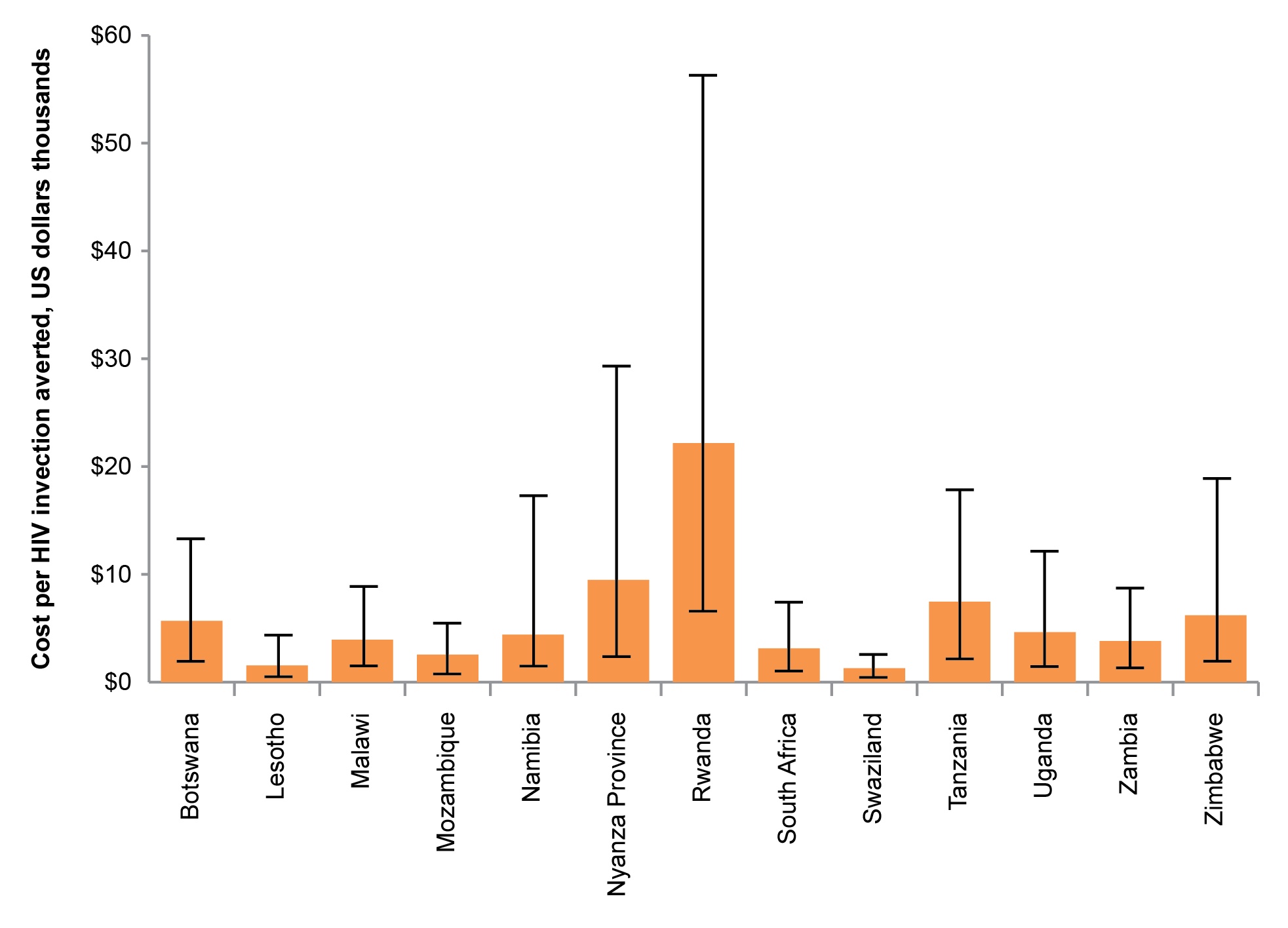

Supplement: S2 Fig — Lower error bars for each country are derived from a model employing the lower uncertainty bound for HIV incidence and the unit cost multiplied by 50%. Upper error bars are derived from a model employing the upper uncertainty bound for HIV incidence and the unit cost multiplied by 150%. HIV incidence uncertainty methods are described in S1 Appendix. (TIF) [file pone.0158767.s010.tif]
